# Co-designing personalised self-management support for people living with long Covid: The LISTEN protocol

**DOI:** 10.1371/journal.pone.0274469

**Published:** 2022-10-11

**Authors:** Celayne Heaton-Shrestha, Anna Torrens-Burton, Fiona Leggat, Ishrat Islam, Monica Busse, Fiona Jones

**Affiliations:** 1 Centre for Applied Health and Social Care Research, Faculty of Health, Social Care and Education, Kingston University and St George’s, University of London, London, England, United Kingdom; 2 PRIME Centre Wales, School of Medicine, Cardiff University, Cardiff, Wales, United Kingdom; 3 Centre for Trials Research, School of Medicine, Cardiff University, Cardiff, Wales, United Kingdom; 4 Bridges Self-Management, London, England, United Kingdom; Purdue University, UNITED STATES

## Abstract

**Background:**

Long Covid is recognised as a complex condition characterised by multiple, interacting and fluctuating symptoms which impact everyday life in diverse ways. The extent of symptom clusters and variability supports interventions that can accommodate heterogeneity, such as personalised self-management support. This approach is also advocated by people living with long Covid and guidelines published by the UK’s National Institute for Health and Care Excellence. Long Covid Personalised Self-managemenT support co-design and EvaluatioN (LISTEN) is one of 15 research projects funded by the UK’s National Institute of Health Research long Covid research programme. LISTEN aims to work with people living with or recovered from long Covid to co-design self-management resources, and a training programme for rehabilitation practitioners to deliver personalised support. The intervention will focus on people not hospitalised for Covid. The protocol presented here details the co-design of the LISTEN intervention which, on completion, will be evaluated in a randomised controlled trial.

**Methods:**

The study will utilise an Accelerated Experience-Based Co-Design approach, and involve 30 people from England and Wales with lived experience of long Covid, and 15 rehabilitation practitioners living with, or supporting people with, long Covid. Through online meetings, participants will share their stories of long Covid, their challenges and strategies to live better with or recover from long Covid, their priorities for self-management resources and the practitioner training andcreate, review and refine these resources and the training. Throughout, LISTEN will draw upon the UK standards of public involvement in research.

**Discussion:**

If effective and cost-effective, the intervention will be available across the UK’s National Health Service. The first of its kind, this study could make a difference to the lives of people with long Covid. To ensure impact, we have developed strategies to involve people from diverse backgrounds and mitigate potential barriers to involvement.

## Introduction

Long Covid is the name collectively given by the community known as ‘long haulers’ in some parts of the world [1] to the complex symptoms which persist after contraction of the Covid-19 virus. Typical symptoms include, but are not limited to, fatigue, joint or muscle pain, altered smell and/or taste, cognitive impairment, anxiety and sleep disorders. A comprehensive study reported a total of 203 symptoms across 10 organ systems [2]. In October 2021, the World Health Organisation (WHO) produced the first official definition of ‘long Covid’, recognising it as an episodic condition with multiple clusters of symptoms [3].

Long Covid is estimated to affect at least 10% of individuals with a positive Covid-19 test, although this is likely an underestimate as many people were not tested early in the pandemic [4]. The potential for a legacy of long Covid is serious, with a high incidence of individuals experiencing symptoms for more than 28 days, not returned to work by six months, and continuing to experience significant symptom burden [2]. In the UK alone, an estimated 1 million people were living with long Covid as of September 2021 [5].

The uncertainty and confusion surrounding long Covid, with its varied, relapsing and remitting symptoms, has been heightened by a heavy sense of loss and stigma [6], and the lack of clear diagnosis in long Covid increases the risk of individuals with the condition feeling overlooked and misunderstood by healthcare professionals and services [7]. Reports of individuals being dismissed as ‘anxious’ while presenting wide ranging and serious symptoms is concerning, given that the medical community’s understanding of the long-term consequences of Covid-19 and access to timely and adequate care is still evolving [3].

While long Covid services are in existence already, for example, in England, the NHS long Covid clinics set up at the end of 2020 [8], these have been highly variable and have not been fully evaluated. Furthermore, to date, no single intervention has shown evidence of effectiveness and, while the NICE guidelines have recommended access to self-management support for individuals with long Covid, the underpinning rationale or approach is unclear.

The Long Covid Personalised Self-managemenT support co-design and EvaluatioN, or LISTEN project was launched in July 2021 against this background. LISTEN will draw on existing evidence from Bridges Self-management (Bridges), a programme theoretically grounded in self-efficacy and underpinned by co-production and co-design [9]. Bridges delivers training and coaching to healthcare teams and focuses on the skills, attitudes, and knowledge of practitioners, transforming therapeutic relationships with patients and facilitating collective change within teams and service pathways [10, 11]. Evidence from research and quality improvement projects has shown Bridges impact on the confidence and skills of patients, and changes in attitudes and practices of practitioners across different healthcare pathways. In 2013, Bridges became a social enterprise that runs in partnership with St George’s, University of London, and Kingston University, and has worked with hundreds of healthcare teams, patients, and thousands of practitioners across the UK, Europe, and New Zealand [12].

The LISTEN intervention draws on recent evidence that relatively short self-management interventions can afford positive outcomes, contrary to the principles underpinning many rehabilitation interventions, namely, that ‘more is better’ [13, 14], and that interventions that rely on signposting and information-giving, which can be the case for long covid (e.g. [15]) have limited evidence of effect [16, 17]. LISTEN also incorporates emerging long Covid evidence. This includes, for example, a growing awareness that a ‘one-size fits all’ graded exercise programme is unlikely to be of use [18], and a briefing paper from World Physiotherapy [19] that supports personalising treatment approaches which focus on recognition and validation of patient experiences.

Additionally, LISTEN is guided by the knowledge that long Covid surveys have tended to miss seldom-heard groups and individuals who have neither received a positive Covid test nor presented to NHS services [2]. Members of long Covid online support groups who have taken part in research on long Covid have generally belonged to middle and upper income groups [e.g. 4] and there is evidence that survey respondents are from similar income brackets [2]. The extent to which current understandings of long Covid, and specifically its impacts and strategies to manage the condition, have included the experiences of people of diverse abilities and people of Black, Asian and Minority Ethnic backgrounds, is unclear. These groups have been among the most impacted by Covid [20] and therefore reaching out, and involving them in developing interventions is critical to ensure that these will not lead to further marginalisation [1].

This project will adopt a multi-staged, co-design approach based on participatory methods to work with more than 45 participants living with, or supporting people with, long Covid. Together, researchers and participants will co-design an intervention comprising one-to-one personalised self-management support from trained rehabilitation practitioners. By harnessing the ideas, solution and priorities of people living with and recovered from long Covid, LISTEN will address the gap that can exist between interventions and the people they seek to support [21, 22]. Finally, LISTEN will work closely with Diversity and Ability, an award-winning disabled-led social enterprise that supports organisations and social justice projects to create inclusive cultures. Through this partnership, LISTEN will develop strategies to include under-served and seldom-heard populations, and ensure the intervention is accessible to a wide range of groups.

This paper describes the protocol for the co-design of accessible and applicable digital and paper-based self-management resources for individuals with long Covid, and training for community rehabilitation practitioners to deliver remote one-to-one personalised self-management support.

## Methods/design

### Study design

The intervention will be developed through an accelerated form of Experienced-Based Co-Design (or AEBCD) [23, 24]. Experienced-Based Co-Design (EBCD) has been used in relation to a wide variety of health conditions and services [23], and involves the active involvement of both service users and professionals to improve the overall quality of these services, including service user experiences [25]. This collaborative process involves gathering patient experiences, for example through observations and filmed narrative interviews (e.g. [26]), summarising the key themes and subsequently using these to trigger discussions around priorities for change. These discussions take place over the course of several months, in the context of facilitated meetings of professionals and patients, both separately and in mixed groups. An accelerated form of EBCD has been shown to deliver a similar impact to EBCD without the development of trigger materials, using instead existing video or audio resources, whether from national archives or a parallel study, and progressing straight to a joint meeting without separate staff and patient co-design events [24, 26].

The LISTEN study will follow a modified AEBCD methodology. LISTEN will hold separate co-design meetings for practitioners and people living with long Covid, followed by a joint event. Co-design participants will share their stories of living with long Covid during the course of the co-design group meetings rather than before the meetings, and their narratives will be used as a trigger to initiate discussion about priorities for the resources and training materials. This will enable the LISTEN team to complete the co-design within a reduced timeframe, that is, within five months rather than the nine to 12 months usually required for a full EBCD cycle.

### Setting

Co-design activities will be held over a virtual platform (i.e., Zoom). All participants will receive a sheet with detailed, accessible, guidance prepared by Diversity and Ability on how to access Zoom meetings together with their ‘welcome pack’. For participants who cannot access virtual platforms or the internet easily, a phone call will be offered so that they may contribute to the co-design process through a one-to-one conversation.

### Participants in co-design

#### Individuals with experience of long Covid

Participants will be eligible to take part in the co-design if they are living with or recovered from long Covid (self-identified), are aged 18 years or above at the time of the co-design, an English or Welsh speaker, or have access to someone who can act as a translator. Residents from any part of the UK are eligible. The Participant Information Sheet makes the eligibility criteria clear but no checks will be carried out to ensure participant eligibility With people reporting disbelief and lack of diagnosis from healthcare providers, people with self-identified long Covid will be recruited to ensure dissatisfactory healthcare experiences do not act as a participation barrier. A further requirement will be that participants do not also take part in the later trial of the LISTEN intervention.

#### Rehabilitation practitioners

Rehabilitation practitioners will be eligible if they work in community rehabilitation services as allied healthcare professionals, nurses or support workers, have experience of supporting people with long Covid symptoms, or have personal experience of long Covid. For instance, rehabilitation practitioners can contribute their experiences of long Covid from a provider and/or service user perspective. Other healthcare professionals (e.g., medics) will not be recruited due to the community rehabilitation focus of the intervention.

### Sampling and recruitment

Our recruitment strategy and sampling will be informed by the diverse population of people living with and recovered from long Covid. We will use a purposive sample to engage and include people with diverse backgrounds, age, gender, ethnicity and ability. All recruitment will take place outside of the NHS, by means of short posts on social media (Facebook, Twitter) and longer information sheets disseminated through local and national long Covid support groups. We will work closely with Diversity and Ability to ensure that recruitment materials are accessible in format and language, and draw on their networks to engage seldom-heard and marginalised groups, including people with existing disabilities, or from minority ethnic communities. Recruitment from seldom-heard and marginalised groups will be monitored and additional targeted efforts will be used to enable wide representation from different marginalised communities.A dedicated email contact will be publicised through social media and other channels.

### Procedure

**Fig 1:**
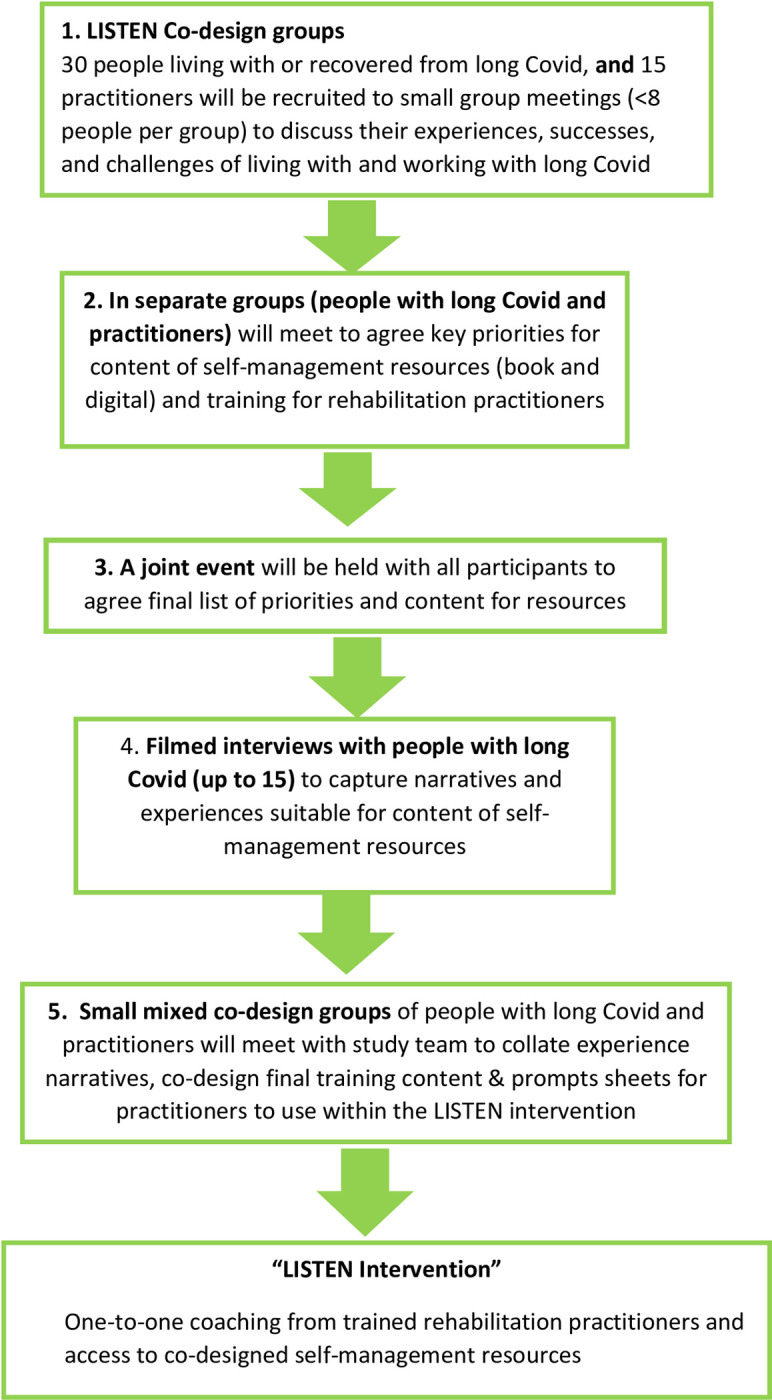
Study flow chart.

Consent processes will enable participants to opt in and out of one or all aspects of the co-design activities outlined in Fig 1 and detailed below.

#### Stage 1: Bringing together people living with or recovered from long Covid and rehabilitation practitioners in separate events (small co-design group meetings)

Initially, we will hold separate co-design group meetings for rehabilitation practitioners, and people living with or recovered from long Covid. Whilst we acknowledge that some of the rehabilitation practitioners will have lived experience of long Covid and people living with / recovered with long Covid may also be rehabilitation practitioners, we make the distinction to emphasise that the two co-design groups will have a slightly different focus. In the two separate groups, we will ask participants to draw, respectively, on: 1) the experience of living with long Covid, and 2) the experience of supporting others, professionally, who are living with long Covid. Separate groups will be hosted to ensure people with long Covid could express their stories without fear of judgment and disbelief from practitioners, and to allow for the development of distinct patient and practitioner priorities.

Ahead of the events, participants will be sent a ‘welcome pack’, which will include a series of questions to guide discussion during the online event, both electronically, by email, and in hard-copy, through the post. This will include a copy of a self-management book for people who have experienced major trauma, stroke or brain injury, as an example of a co-designed resource developed by Bridges self-management [9].

Each online co-design group will include up to eight participants, to ensure all members have the chance to tell their story, to discuss the challenges they faced as people living with long Covid and the solutions they developed to live better with long Covid (and, for some, to recover from the condition). Building on what they have shared and discussed with other group members, and a presentation about the Bridges book, the group will then begin exploring their ideas about, and priorities forthe contents of the self-management resources for people with long Covid. They will also reflect on the main messages for rehabilitation practitioners arising from the group’s discussions.

Group meetings will last up to two hours, including time for a pre-meeting ‘meet and greet’ session, where participants can chat informally, and a break half-way through the meeting. Participants will be reminded that they can take further breaks as needed during the meeting.

A minimum of two people with expertise in co-design will facilitate the meetings, drawing on a variety of strategies to generate ideas from the groups. Group discussions will include access to a virtual white board, break-out rooms and interactive activities to generate discussion. Meetings will be recorded for the purpose of summarising key experiences. A meeting summary will then be sent to all participants and their reflections on the summaries sought, in preparation for the larger mixed co-design group meeting. Those with no internet access or who are uncomfortable with the online medium will be offered the option of contributing to the co-design through a telephone conversation with a member of the study team. Their contribution will then be included within the summaries sent to participants for their feedback. All summaries will be checked for accessibility, following guidance provided by Diversity and Ability.

#### Stage 2: Bringing together people living with or recovered from long Covid and rehabilitation practitioners in a join event (large co-design group meeting)

Participants from all initial small-group meetings will come together in a large mixed-group meeting of 30–45 people (both practitioners and people living with or recovered from long Covid), also lasting up to two hours and with an informal pre-meeting session and mid-way break. In this session, participants will have the opportunity to share the topics discussed during the initial small group meetings. Feedback on priorities for the LISTEN intervention will be discussed in small breakout rooms to allow each person’s voice to be heard by others. The joint event will be recorded but not the dialogue in breakout rooms. Instead, participants will be invited to give a verbal summary from their breakout room discussions. A final summary will be developed by the study team including the priority list and sent out to all participants for feedback. For participants who do not have internet access or are uncomfortable with the online medium, we will again ask if they wish to contribute by means of a telephone conversation with a member of the study team.

#### Stage 3: Conducting narrative interviews with people living with or recovered from long Covid

Interviews will be conducted with up to 15 people living with or recovered from long Covid, selected among participants in previous co-design meetings who have consented to taking part in a filmed interview. The 15 participants will be selected to reflect the diversity of participant characteristics, including symptom type and severity and also where they feel they are in their long Covid ‘journey’. For example, while there may be no singular recovery ‘journey’ or trajectory, stages are likely to include recent development or diagnosis of long Covid, living with and managing the condition for a while, experiencing a relapse, or fully recovered. These interviews will be used to generate content for the LISTEN intervention book in hard-copy and digital form and, in addition, used within practitioner training.

Semi-structured interviews will be guided by an initial opening question encouraging the interviewee to tell their story of living with long Covid, the challenges they encountered and the solutions they developed. Following the opening question, the member of the study team conducting the interview will only use occasional prompts to keep the narrative flowing and on topic (when it is felt appropriate to do so). The interviews will be less than 60 minutes in length and participants will be given the option to do their interview over several separate sessions to help manage fatigue and difficulties with concentration. Participants will also be able to cancel and re-schedule interviews at short notice should fluctuating symptoms prevent participation, and will be sent the interview topics at least one week in advance to allow for any preparation. Each interview will be carried out and recorded using Zoom, then transcribed and anonymised.

#### Stage 4: Co-design work in small groups focusing either on the contents of the resources or training

Participants in the earlier co-design meetings will be invited to a further small co-design group meeting of up to two hours, following the format of the previous group meetings, and including both people living with or recovered from long Covid and rehabilitation practitioners. In one meeting participants will work together to refine and finalise the content of the self-management resources. In the other meeting, participants will focus on finalising the training for practitioners. These two groups of up to eight people each will collate of a ‘bank’ of narratives by people living with or recovered from long Covid in filmed and written form, and agree on the content. For participants who do not have internet access or are uncomfortable with the online medium, we will again ask if they wish to contribute by means of a telephone conversation with a member of the study team.

#### Stage 5: Finalising the resources, training and coaching: The LISTEN intervention

An interative approach will be used in working with co-design groups to develop the book in hard-copy and digital form, and the training for rehabilitation practitioners.

The book will avoid technical language and will be written through the experiences and words of people living with long Covid. We will work closely with a design company, and Diversity and Ability to ensure the layout and font size is accessible and the sections are easy to navigate. The order and content will be agreed and refined by the research team and the co-design group.

Training for rehabilitation practitioners will draw on existing training methodology developed by Bridges self-management but will be refined to include key examples, preferences and content generated from the co-design stages. Trained rehabilitation practitioners will be supported to deliver the intervention by the Bridges self-management team according to key fidelity markers. We anticipate this will comprise one-to-one personalised self-management support delivered remotely over six sessions within a 10-week timeframe. The fidelity of intervention delivery will be evaluated as part of an extensive process evaluation within the definitive trial. Practitioners delivering the intervention will become a community of practice with regular coaching support from the Bridges team and a clinical psychologist, and access to podcasts, webinars and prompt sheets.

### Patient Public Involvement and Engagement (PPIE)

Public and patient involvement and engagement is a central element of LISTEN, and more particularly the co-design phase of the study. Supporting us in developing and refining our PPIE are Kingston University’s Centre for Public Engagement [27] as well as the PPIE leads and panel in the NIHR Applied Research Collaboration South London (NIHR ARC South London) [28].

In developing our PPIE plan, we have complied with the guidelines set out in the UK Standards for Public Involvement [29]. The UK Standards for Public Involvement were developed over three years by a UK-wide partnership including representatives from the Chief Scientist Office (Scotland), Health and Care Research Wales, the Public Health Agency (Northern Ireland), the National Institute for Health and Research (England) and independent experts, and were pilot-tested by 40 individuals and institutions during 2018–2019. The guidelines describes what ‘good PPIE’ looks like, and seeks to encourage approaches and behaviours that characterize good public involvement in health and social care research. The six standards are intended to facilitate the development of such approaches and behaviours, and reassure both researchers and the public that best practice is being followed in this regard.

### PPIE planned actions

A matrix comprising the six UK standards of public involvement and the different stages of the LISTEN project will be used to record the planned actions and log actual actions relevant to PPIE throughout the project’s life. To enhance the quality of the project and to ensure the lived needs of people with long covid are addressed, the LISTEN project has planned to action these standards as follows:

#### Standard 1 inclusive opportunities

The inclusive opportunities standard refers to the provision of public involvement opportunities which are accessible and reach people and groups according to research needs.

To involve people living with long Covid and rehabilitation practitioners from diverse backgrounds and seldom heard groups, we will work with Diversity and Ability as previously described. We have contacted long Covid support groups such as Covid:aid [30], and Long Covid Support [31], and other health and inclusion focused organisations such as Health Watch England [32] and National Voices [33] in order to reach people specifically concerned by this research. Throughout the process we will continue to engage with the long Covid community in various ways.

We will ensure opportunities for involvement are accessible by providing multiple options and supportive guidance, including guidance on accessing and joining the co-design meetings. Where internet accessibility may act as a barrier, alternatives to online meetings will be offered (e.g., talking to a researcher on the phone). To develop rapport and enhance participant comfort, short informal discussions will be offered to potential participants prior to joining the co-design meetings. Diversity and Ability will help access interpreters for people who would like to participate, but do not have English as their first language. Honoraria will be provided to members of the public who work with us during the co-design study.

We will use a range of approaches to introduce and update the study activities, including social media (e.g., Twitter) and a blog. The blog and the twitter page will invite comments and feedback from the public on the study.

#### Standard 2 working together

Working together refers to a way of working which values all contributions, and that builds and sustains mutually respectful and productive relationships.

To work with the public in the design of the research, we set up a PPIE advisory group of people living with long Covid. The group will meet every two months during the co-design study, and quarterly thereafter until the end of the LISTEN project (August 2023). The group will be facilitated jointly by the LISTEN Chief Investigator and a healthcare professional living with long Covid.

We will also work together with the public during the co-design stages. We will draw on the Bridges self-management methodology which prioritises diverse voices, needs and experiences of individuals and families to (a) agree key design areas and set priorities; (b) initiate the co-design of training materials, digital resources /paper-based workbook; (c) co-design content, prompts and brief guides for delivering the intervention; and (d) co-deliver training.

Several strategies will be employed to ensure we work together in a mutually respectful and productive way. Firstly, co-design activities will be jointly facilitated by the researchers and the Bridges team. Two or three individuals with experience of generating ideas and solutions from groups will be present, supported by Diversity and Ability. Participants will be encouraged to share as little or as much as they wish. At the outset of the activities, the research team will describe their roles and emphasise their desire to listen and work with people to achieve a mutual goal.

Secondly, provisions will be made to manage the physical and cognitive symptoms of long Covid and allow participants to contribute as fully as possible. We willinclude breaks within sessions, opportunities to turn cameras off, encouragement to use the chat and response functions instead of verbal interaction, and sending out summaries after each session to allow for further reflection at a time that suits participants.

Feedback from the co-design participants will directly inform the development of self-management resources and the practitioner training. The process will be iterative in direct response to participant suggestions logged during and after meetings, and should contradictions arise, these will be discussed until a mutual decision is reached. Feedback from the PPIE group meetings will be summarised and circulated for further reflection. Ideas to take forward will be taken to research team meetings, and if any concerns are raised, these will be logged and reported to the study chief investigators.

Finally, all PPIE group members will be remunerated for their time attending meetings according to guidance published by NIHR [34], and engaging in any additional activities (e.g., meeting preparation, providing feedback on materials). Co-design participants will receive vouchers for their participation in different stages of the co-design process.

#### Standard 3 support and learning

The third standard relates to the promotion and offer of support and learning opportunities that build confidence and skills for public involvement in research.

Using our links with the PPIE leads of the NIHR ARC South London, we will offer support and training to all members of the PPI advisory group, we will offer support and training to all members of the PPIE advisory group. The extent of training required and the mode of delivery will be decided through discussion during our first group meetings. We will also have access to support for our PPIE group from the Centre for Public Engagement in Kingston University’s Faculty of Health and Social Care.

During co-design activities, people living with long Covid and rehabilitation practitioners will be offered the opportunity to design and deliver previous meeting summary presentations. The research team will offer support where requested to enhance participants’ skills and confidence to create and deliver short presentations, and to promote better engagement in the research.

#### Standard 4 communications

This standard encourages research teams to communicate in ways that are relevant, accessible, well-timed and effective.

To ensure that communication is relevant and accessible, we will work closely with Diversity and Ability who will review our communications, recruitment and activity materials for accessibility, and guide our overall approach. In recognition of the physical and mental symptoms of long Covid, co-design activities will not occur more than once per week. Individuals involved will be contacted via email about the co-design activities, and materials for the activities will be sent in advance to allow for preparation.

#### Standard 5 impact

Impact refers to the improvement and difference that public involvement makes to research

The involvement of the public in LISTEN, and particularly in the co-design phase, is integral and without it, the study would not take place. The LISTEN PPIE advisory group will guide us throughout the study and provide input into the co-design of the LISTEN intervention. Note-taking during meetings and keeping records of meeting discussions will allow for the monitoring of public involvement in the overall study and facilitate periodic reviews of public involvement. Co-design meeting summaries will enable us to track how ideas were proposed in the small co-design activities, developed in future activities, and represented in the final intervention.

We will include a discussion of the difference that public involvement makes to research as part of the LISTEN management group meetings, and the involvement of the public in the LISTEN study will be made explicit in any research outputs such as reports and publications, though individual identities will be kept confidential. At the end of the co-design phase, we will ask people with long Covid and rehabilitation practitioners to share their experiences of research involvement and describe how they feel that involvement impacted them personally.

We will co-determine outcome measures to assess the impact of the PPIE for all six standards across the relevant stages of the trial.

#### Standard 6 governance

Governance refers to the involvement of the public in research management, regulation, leadership and decision-making. We will provide various PPIE opportunities in the formal research governance of the LISTEN project. One PPIE representative has been named as a co-applicant on the project funding application. A further PPIE representative will be included in the project management group and their active participation encouraged. This group will meet monthly to discuss key management issues and monitor project progress against stipulated milestones.

The LISTEN PPIE advisory group will provide further involvement opportunities. When the co-design phase of LISTEN ends, participants in the co-design will be invited to join the LISTEN trial PPIE advisory group, allowing the PPIE group to expand, benefit from more voices and experiences, while also allowing practitioners and people with long Covid to get involved in research management and decision-making.

### PPIE reporting

To report PPIE formally, we will be following the GRIPP2 short form guidelines [35]. GRIPP2-LF (long form) and GRIPP2-SF (short form) are the first international evidence-based guidance for reporting patient and public involvement in health research, developed through three rounds of Delphi questionnaires of over 140 experts in the field. The long form comprises 34 items and is best suited for projects that aim to develop PPIE specifically, while the short form includes five items (aims, methods, results, outcomes, and critical perspective) and is suitable for all other types of projects.

Consistently, LISTEN will report against these five items by: outlining the aims of PPIE in the study; describing the methods used for PPIE in the study; detailing the results of PPIE in the study, including both the positive and negative outcomes if applicable; comment on the extent to which PPIE influenced the study overall, including both positive and negative effects where this applies; and reflect on the LISTEN experience with PPIE critically, highlighting what worked well or what worked less well.

### Discussion

The current study aims to co-design the LISTEN intervention for non-hospitalised people living with or recovered from long Covid, reflecting the ideas, experiences and narratives of this group of people. This will progress to an individually randomised controlled trial (REC reference number: 21/WA/0368; IRAS reference number: 306220) to evaluate the clinical, and cost effectiveness of the personalised self-management support intervention compared to usual care as currently available in the NHS. Alongside, a mixed-methods process evaluation will inform an integration and sustainability package that could be subsequently used (should the intervention be effective and cost-effective) as part of a rapid national scale-up effort through the UK’s National Health Service (NHS).

We anticipate several possible challenges to the co-design study: firstly challenges tied to the choice of the online medium to recruit participants and run the co-design meetings, and secondly, to the management of long Covid symptoms among participants. We have developed a number of strategies to mitigate these challenges.

### Accessing the co-design group meetings

All co-design group sessions have been planned on a virtual teleconferencing platform (Zoom), both to minimise ongoing risks to participants arising from the Covid-19 pandemic and also to enable the study to include participants from a wide geographical area. This has practical implications in terms of access. For some participants the use of Zoom may be completely novel thus leading to problems with login or using the Zoom platform features. A broader issue will be relying on participants to have quality internet access in order to access the co-design meetings and review any materials sent electronically. While we are not able to control factors linked to the quality of internet, we have sent all participants accessible guidance (developed by Diversity and Ability) on using Zoom. We will also offer the option of contributing to co-design through a telephone conversation where participants either do not have access to Zoom or do not feel confident in its use.

### Recruiting participants to the co-design meetings

Our reliance on online methods of recruitment (social media, email) has the potential to exclude individuals from seldom heard groups, who may not engage with these platforms regularly or not have regular internet/email access. Therefore there is a risk of missing a vital sample of individuals who would be suitable for the study and would also be willing to participate. The research team will closely and regularly monitor the profile of co-design group participants and will encourage those with a social media presence to draw attention through other methods (e.g. ‘word of mouth’) to our request for public involvement from communities that have not been represented in co-design meetings till that point. We will be flexible in the scheduling of online meetings to accommodate participants from harder-to-reach groups, and offer phone conversations rather than online meetings if preferred.

### Fatigue as a barrier to involvement in co-design

Individuals with Long Covid often experience fatigue which may be exacerbated by mental exertion. The length of the co-design meetings as well as the use of the online medium, may dissuade potential participants from coming forward and also lead to drop out from the study.

In order to address this issue, we have made provision for a break during the co-design meetings and emphasise that participants may take further breaks as required and/or turn off their cameras. In addition, we will indicate in the ‘welcome pack’ sent to participants and again in co-design meetings, that participants may opt in or out of meetings as they wish. Similarly, we will offer participants in the filmed interviews the option of conducting the interview over several sessions. Finally, by inviting participants to add to the meeting summaries after each meeting, we will provide an opportunity for participants whose symptoms made contributing during meetings challenging, to contribute more fully. Nonetheless, this means that we cannot guarantee the same sample of participants throughout the study and may need to recruit more participants to obtain the number of participants required.

The LISTEN co-design study is now underway, in full compliance with the protocol outlined in this paper. It is expected to complete at the end of February 2022, when the trial of the LISTEN intervention will begin, together with the process evaluation, and continue until August 2023. Starting with this protocol, the LISTEN team will document and endeavour to make public the procedures, achievements and results of the LISTEN project, ensuring transparency and quality of the study, and ultimately, a more impactful contribution to current approaches to the treatment of long Covid.

## Supporting information

S1 File (PDF)

## List of abbreviations

AEBCD: Accelerated Experienced-Based Co-Design
EBCD: Experienced-Based Co-Design
