# Supplementary material for: Co-designing personalised self-management support for people living with long Covid: The LISTEN protocol
Source: PLoS One. 2022 Oct 11;17(10):e0274469. doi: 10.1371/journal.pone.0274469 (PMC9553035; doi:10.1371/journal.pone.0274469)
Supplement: S1 File — (PDF) [file pone.0274469.s001.pdf]

LISTEN co-design group meeting 18th November 2021

Large Meeting Summary

Similarities and differences within the small group summaries

Presentations given by each of the four small co-design groups.

Similarities across the groups included:

- **Physical and psychosocial challenges** - fatigue, brain fog, loss of identity, isolation/ abandonment, fear, shame, uncertainty, guilt, managing other's disbelief

- Tips for navigating everyday life:
    - Acceptance that **recovery is not linear** - ups and downs are normal and you should be ready for those to happen
    - Importance of **finding positives** – try to find positives in the situation and think about what you might have gained, rather than lost
    - **Rest, pace, and plan** – budget your energy, and plan for cognitive and physical rest after activities
    - Focus on and **prioritise you** – put your needs first and try to ignore the disbelief of others who may not understand
    - **Ask for help** and **seek medical reassurance** – finding out the condition of your hardwiring and physiology can help you to feel more comfortable
    - **Monitor** the journey – record your ups and downs, and your symptoms so you can see the progress; records/diaries can be useful during appointments with health services

- Ideas for resources and training:
    - Provide space (resources) and time (training) for **offloading and monitoring** the journey
    - **Variety of stories** from variety of people – include people from different phases in the journey (e.g., early on, nearing their normal), different walks of life (e.g., profession/family), and different demographics (e.g., age, gender)
    - Advice and **recognition of the challenges** – normalise & validate the psychosocial struggles.

Differences between the groups included:

- Tips for navigating everyday life:
    - Plan for **joy** – purposively build in something into each day which brings joy
    - **Celebrate stability** – treat the plateaus in symptoms and the journey as successes; maintaining a level of activity is progress
    - **Experiment** with advice – try new things and find what works for you

- Ideas for resources and training:
    - **Language** cautions – be aware of the stigma surrounding 'self-management' and the implication of 'recovery'

  - Support for engaging in **challenging conversations** with family/friends & medical professionals
  - Bite-sized, safe information – a **first-aid kit** for quick strategies; ideas to experiment with that won't cause harm; **medical jargon** to navigate services.

What needs to be in the book?

- An **introduction which sets the scene** – very articulate introduction to **manage expectations** of what the book is for (e.g., not a medical go-to); give permission for people to think outside the box; state it is an unknown context where not all the answers exist.

- **Variety of stories**
  - From people with different symptoms, and from different backgrounds (e.g., family/sporty/older/profession)
  - From people at different points in the journey; stories of recovery or which end on a positive and present hope.
  - From people with other illnesses/co-morbidities to show how symptoms may present differently and pose different challenges

- Normalisation and **validation of both physical and mental symptoms** and their interconnectedness.
  - Descriptions of what these symptoms feel like so people can relate – fatigue is more than just tiredness
  - Give range of symptoms not just the most common (e.g., brain fog/fatigue)

- Normalisation of **relapse and non-linear journeys** – prepare people for relapse and emphasis that relapse is not failure; provide ideas for re-building after relapse

- **First-aid kit** for quick strategies/hint and tips - coloured coded to clusters of symptoms; not gold-standard treatment, bit real-life strategies

- **Activity building blocks** (e.g., from getting dressed to exercise) – loose structure/ideas for how to build activity back up; re-frame exercise as activity; emphasise this is not prescriptive

- Links to **current medical/healthcare advice** – diet guidance; medical information; NICE guidelines; breathing exercises; clinical treatments

- **Support for navigating health services** – table/space for recording appointments, medication, tests/screenings, and symptoms; types of tests/screenings which may be suitable for different symptoms and reassurance

What needs to be in the training?

- Emphasis and **explanation of patient-centred care** and **individualised care:**
  - Describe what is meant by co-production and the power of it.
  - Show **how solutions can be co-produced** with patients – remove fear of doing it.
  - Co-produce a bronze, silver, gold scheme based upon time, so solutions can't fail
  - Give patients permission to work with you to come up with ideas

- Normalisation of **being comfortable with the uncomfortable** - it's okay to not know the answer as there may not any; accept that trial and error is a useful solution

- Development of **language skills** – support with articulating language which is **free of judgment**; develop confidence and comfort with using language; develop language which is accessible and useable; practice using language to develop **authenticity** and avoid patronisation.

- Development of **listening skills** – support with developing active listening.

- **Preparedness for the evolving nature** of the condition – long Covid will be unpredictable, and symptoms will constantly change and fluctuate

Additional considerations for the training & resources:

- **'Any other business'** is important – not prescriptive; give permission for people to share things that are new/unique.

- Striking a **balance between acceptance and hope** – normalise and validate that life and living with long Covid might be challenging, but also include aspects of hopefulness and show that things can get better.
